# Control Work

**Published:** 1896-02

**Authors:** 


### CONTROL WORK.

*Connecticut.* At Pomfret, thirty-eight out of a herd of fifty-two Jerseys were condemned by the tuberculin-test, and with very few exceptions proved tuberculous. These examinations were made in the field at the time of destruction, and was not as thorough as they might have been.

The *Telegram*, of Worcester, Massachusetts, says that the efforts to raise a fund of money to fight the Cattle Commision this winter in Boston has met with very little success. Many of those who most bitterly opposed the Commission a year ago are not only friendly to it now, but milk producers are realizing the necessity of assuring their customers of the healthiness of their herds and sanitary condition of their dairies. The public have been aroused to the necessity of demanding that they be insured a wholesome supply of milk. Several farmers who have recently purchased new cattle have had them all tested before bringing them into the State.

Mr. George Austin Bowen, of Woodstock, a believer in the efficacy of tuberculin, at a meeting of the State Grange at Athol, Massachusetts, came out victorious in a controversy on the subject, and the more important officers of the Grange elected are advocates of a thorough eradication of tuberculosis in Massachusetts.

The annual report for the year ending December 15, 1895, of the Massachusetts Cattle Commission has these very salient features incorporated therein. The list of inspectors in the various towns seems to have almost every occupation represented among the number except a preacher. The undertaker, the barber, the butcher, the fish-monger, horsedealer, janitor, etc. This is one of the weak features of the work, and should be remedied; it is one of the infamous results of the miserable spoils system of politics under which we live. These men are charged with the duty of meat inspection, slaughter-house supervision for contagious and infectious diseases, and it will be readily seen how imperfect such a system must become under such a class of inspectors.

No tuberculosis has been found in sheep—frequently noted in swine.

The law of 1895 militated against a reliable inspection very much, leaving it to physical methods when so demanded by owners and farmers.

Lack of funds are retarding the work very much, and the quarantine expenses are thus made greater. Since October inspections have been carried out in 69 towns, examining 4769 herds, with 26,756 cattle, 5412 sheep, and 13,061 swine. Between June 5th and December 15th, 18,938 cattle, 36,720 sheep, and 2779 swine were inspected at slaughter houses. Under the same law 503 cattle, 212 sheep, and 1034 swine have been inspected as killed by owners; the meagre character of such returns indicate a non-compliance with the law. Some 192 cattle, 8 sheep, and 97 swine proved tubercular. In regard to the sheep the Commission say the opinion as given was by inspectors and is unsupported by laboratory investigation. Two hundred and six cases of glanders were destroyed, and autopsy corroborated the diagnosis made. This is 46 more than were killed in 1894. The Board points to common watering places as a potent source of its perpetuation. No less than 65 outbreaks of hog cholera are reported. Rabies in but a few towns.

The report reviews the experimental tests at the cattle markets, and a careful examination satisfies the Commission that the errors formerly reported cannot be justly attributed to the failure of tuberculin as a diagnostic agent, but due to local causes of excitement of the animals under inspection, and this work has been abandoned, examinations with tuberculin only being conducted now of animals under normal conditions.

One autopsy revealed tuberculosis of the eyelid; the brain, spinal cord, lower joints of limbs have all been noted as points of attack.

The establishment of interstate certificates of inspection by reputable veterinarians, based upon tuberculin tests, have been accepted, and restricted the opposition and difficulties in the work. Purchasers who demand a certificate of inspection are on the increase, realizing the value therein.

Of the first 793 condemned, 15 failed to reveal on autopsy lesions of the disease. Of the last 1000 condemned, 989 were found diseased; of the 11 in question only 3 autopsies were made by agents of the Board.

The following shows the increase in the number of herds the

Board was requested to examine with tuberculin under the provisions of the law of June, 1895: For July, 10; August, 68; September, 74; October, 92; November 138; to December 16th, 153 requests. These requests involved the examination of 4093 animals, of which 1081 were condemned. In 2 cases on autopsy the disease was not found, a fraction of 1 per cent. in error. These herds were dairy stock, and physical examination would have been fruitless, and suspicious animals to the eye were rarely noted. Of the 314 herds thus tested, only 24 were found free from the disease, a percentage of 26.3; in New York State, under similar conditions, the percentage was 34.9. Tests made by private veterinarians, covering 584 animals, showed 90 condemned, only 1 of which failed to reveal evidence of the disease. The accuracy of the tuberculin test is fully indorsed; the feeling against its use measurably overcome, and the fear of harmful influence upon healthy animals steadily disappearing.

Average compensation $35 per head for those destroyed. Under the present system many large centres of milk production have been cleaned up, and greater zeal will follow in keeping the disease out in purchasing new animals. The public are being rapidly educated on the question, and their demands so rapidly increasing for evidence of a source of supply, of pure milk that the Board feels that self-protection will rapidly win over those who have objected to the inspection and who fought the systematic plan originally inaugurated. Under this plan the Board do not expect to rid the State of the disease, but only at the most to restrict the dangers and limit the extent and prevalence of the disease. Systematic inspection, thorough disinfection of premises, and rigid quarantine regulations for all fresh cattle brought into the State are the only absolute safeguards in the opinion of the Board.

Riper experience teaches that the contagious principle is stronger than first supposed. Contagion may be quick and rapid, but it is extremely subtle, and its extreme development may be slow and inconspicuous.

Ten per cent. was the original estimate of the Board as to the number diseased in their State. Of those examined it has reached 16 per cent. It is to be said that in a large number of instances the herds were considered suspicious.

The dangers in using the milk and meat of diseased animals, especially milk, are freely commented upon, and the opinions of leading authorities over the world are quoted to support the position of the Board on this point. It quotes the greater frequency of udder lesions than were formerly supposed. They recommend severe form of regulation by which milk shall not be delivered in the State except that coming from animals which have been properly shown to be free from disease.

Referring to the unreliability of physical examinations, it states that during the past year it would have failed to detect the presence of the disease in a majority of the animals tested on voluntary requests for such examinations, while on the other hand tuberculin demonstrated that the disease was not present in more than 55½ per cent. of the animals quarantined on suspicion by the inspectors. The healthy animals are not injured by testing with tuberculin; herds are quoted where they have been tested over and over again without injury. Tested animals are worth more in the market, and thorougbred animals can scarcely be sold without having been tested.

Better sanitary conditions of the stable are strongly urged; the exclusion of human consumptives from stables, though the greater danger is from animals to man, laboratory research proving that the bovine bacillus kills out the human bacillus. Up to June 5, 1895, $91,876 were expended, of which amount $47,500 went to the owners of condemned animals; from June 5th to December 15th, $75,600, $59,500 of which went to owners for condemned animals.

At Whately, in December, a herd of 16 cattle were tested with tuberculin; 14 responded to the test and, were ordered destroyed by the Commission. These were registered Pole and thoroughbred Durham.

Out of 60 cattle tested at Amherst, 17 were condemned, destroyed, and found diseased. Many others are requesting tests of their herds.

The *Mail* of Fitchburg, of December 24th, in an editorial, refers strongly to a demand from the public for milk from tested herds.

Of the herds recently examined at Worcester, 50 per cent. were condemned. The expression of owners who appreciated the necessity of their herds being freed from diseased stock, no matter what the inconvenience. This has led to more satisfactory conditions of stable, where these herds were formerly housed.

The Worcester *Telegram* of December 26th in a leading editorial strikes hard the anti-tuberculin syndicate, which last year succeeded in having removed from the law under which the Commission was working, the compulsory tuberculin test, and points to the fact that the Commission are at present overwhelmed with written requests, many of which are from former antagonists of the Commission, to have their herds tested with tuberculin.

Dr. George N. Kinnell, inspector of cattle for Pittsfield, in making his report to that city, refers very forcibly to the slow and insidious character of tuberculosis, and how uncertain the diagnosis by physical examination, and referring to the absolutely harmless effects of tuberculin as a diagnostic agent, and feeling that a certificate of the healthfulness of the herd made upon physical examination is practlcally worthless, made the following proposition to the local dairymen: That 25 per cent. of the animals in each herd be submitted to the tuberculin test. In the event of none of these animals reacting the balance of the herd should be presumed free from the disease. Should one or more of the 25 per cent. react the entire herd should be subjected to the test forthwith. Condemned animals to be destroyed and paid for by the State. This plan was adopted and applied to all herds except one supplying milk within the city limits, or to the licensed men who offered milk for sale on the street. Number of herds inspected 29; number of animals, 450. Disease was found in 9 herds; total number tested, 258; 33 condemned; valuation of condemned animals was $1000; average valuation, $33 per head.

Governor Greenhalge, in his inaugural address to the Legislature, after referring to the work of the Tuberculosis Commission for 1895, says: "There seems to be less opposition to the work of the Commission and an increase of confidence in the methods adopted. It is true that the whole subject is one of difficulty, and there is need of patience and judgment on all sides in order to obtain the best results. I trust the report of the Commission will receive your most earnest cousidertion."

Pittsfield establishes a unique position in the State, where practically the entire stock has been submitted voluntarily to the tuberculin method of examination. It has found favor among dairymen, and will no doubt be continued in conjunction with the introduction of new cattle.

*New York.*—A meeting of the State Board of Health in New York, in December, showed that since July 15, 1895, 853 head of cattle had been examined; 298 condemned, or nearly 35 per cent.; that the tuberculin test was trustworthy in every case, and how best to protect the milk-consumers was under consideration. President Wilson suggested that the Legislature should enact a law providing for the inspection of all the dairy-herds from which New York city's milk-supply is secured, and Dr. Biggs, director of the city board's bacteriological laboratory, showed that an effective inspection could be made.

The *Times* says that unless the State will take some action there is no question but that the city will take measures to protect its people from the dangers of the milk-supply from such diseased sources.

*Maine.*—Portland's Board of Health, desiring that the city's milk-supply should be from healthy animals, through the Board of Cattle Commissioners, have been investigating the condition of the herds supplying that city. A herd of more than 30 were recently tested, and 18 out of 31 responded to the test. But one case was discovered in this herd by physical examination.

*Vermont.*—Mr. C. M. Winslow, of Brandon, whose herd was freed from tuberculosis once, is again having the herd tested as an additional assurance to his patrons that he is supplying them with a pure article. This plan will become more generally adopted in every locality in the near future.

The same gentleman, speaking on the value of tuberculin, said he considered it the most reliable diagnostic agent ever introduced; without it there was no hope of cleaning the diseased from a herd. It was an extremely delicate test, and should only be used by careful and scientific investigators. The errors charged in its use, he believed, were not the fault of the diagnostic agent, but due to lack of thorough and careful conditions, circumstances and environments at the time of its use. He had no mistakes in those where he had used it, and afterward destroyed the subjects, every one revealing evidences of the disease.

An outbreak of anthrax in a herd in this State, numbering 125, where 14 had died from gross carelessness in dealing with the carcasses, but one death occcurred subsequently to the primary vaccination. It is confidently believed that it will be a safeguard in allowing the use of thousands of acres of infected territory in this State alone.

*Pennsylvauia.*—Philadelphia's Meat-Inspecticn Department connected with the City Board of Health shows that 145,059 cattle were inspected, of which number 162 were condemned and destroyed; 129 of these were suffering from tuberculosis and 33 with other diseases; 87 sheep, 72 hogs, and 280 chickens were condemned as unfit for food; 2297 calves were found to be too young to be killed. Ten persons were also arrested, charged with selling unwholesome meat.

A preliminary meeting of the State Veterinary Sanitary Board was held in January. State Veterinarian Pearson was elected Secretary, and rules for the government of the Board were discussed.

A meeting of the Associated Health Authorities of Pennsylvania convened at Harrisburg in January. Among the subjects discussed were "Legislation for Pure Milk;" "The Sanitary Relation of Slaughter-houses and Pork-packing Establishments; of the collection of a large number of hogs and the various examinations from the process attendant on the commercial utilizatiou of offal to diphtheria;" the law of 1895 to limit the spread of contagious diseases.

Tuberculosis has again developed in the herd of cattle at the Norristown Insane Asylum. Active measures are in operation for its stamping out. We understand no precautionary measures were adopted in reëstablishing this herd, a danger which is too little appreciated.

*Colorado.*—Veterinarian Gresswell reports that the use of anthrax vaccine has proved very efficacious in reducing the losses from this disease. In a number of herds where at times the losses amounted to one or two head per day the disease has entirely disappeared.

*Louisiana.*—The use of vaccine to control black leg among mules has proved very satisfactory.

*Wyoming.*—Experimental tests are in use among a bunch of horses in this State for blaek leg, Pasteur vaccine being used for protective purposes.

*Virginia.*—Anthrax vaccine is being used in a district where the disease has broken out in cattle.

*New Jersey.*—President Rowell, of the Bridgeton Board of Health, reports that 94 herds were infected with anthrax in that district, and of this number 600 cows, 267 horses, and 50 mules were infected, of which 174 cows, 32 horses, and 16 mules died, a total of 222 deaths. Of these, 150 cows, 23 horses, and 13 mules were not inoculated; 17 cows and 2 mules were inoculated with the first lymph, and died before the second inoculation, and 3 cows, 4 horses, and 1 mule died after the second inoculation. Pasteur anthrax lymph was used in this district.

*California.*—The Board of Supervisors of the County of Alameda ordain as follows:

Section 1. Every milch cow or other animal within the County of Alameda having tuberculosis shall at once be deprived of life by the owner or person having charge thereof, upon discovery of its condition, any such owner or owners omitting or refusing to comply with the provisions of this section shall be deemed guilty of a misdemeanor.

Section 2. Any person violating the provisions of this ordinance, upon conviction thereof, shall be punished by a fine of not less than fifty dollars, and not more than one hundred dollars, and in case such fine be not paid, then by imprisonment in the county jail of said County of Alameda at the rate of one day for every two dollars of the fine imposed.

This ordinance shall take effect and be in force thirty days after its passage.

*National.*—The Secretary of Agriculture makes a strong point in his report of the work of the Bureau of Animal Industry, Inspection of Meat Department, when he points to the necessity of State and municipal authorities intelligently and diligently co-operating with the National inspection, and thus spare the people in great killing centres from consumption of rejected animals and meats. He wisely urges the individual to demand the same inspection and certification to the products he purchases.

1,361,800 animals were inspected during 1895, for foreign shipment, of which number 1060 were rejected. Every individual animal was tagged and numbered so as to be easy of identification.

Better safeguards are demanded for the disinfection of cars, stock-yards, and ships, to protect sheep from the development of scap in transit.

